# Two-phase quasi-equilibrium in β-type Ti-based bulk metallic glass composites

**DOI:** 10.1038/srep19235

**Published:** 2016-01-12

**Authors:** L. Zhang, S. Pauly, M. Q. Tang, J. Eckert, H. F. Zhang

**Affiliations:** 1 Shenyang National Laboratory for Materials Science, Institute of Metal Research, Chinese Academy of Sciences, 110016 Shenyang, China; 2 IFW Dresden, Institute for Complex Materials, P.O. Box 27 01 16, D-01069 Dresden, Germany; 3 TU Dresden, Institute of Materials Science, D-01062 Dresden, Germany

## Abstract

The microstructural evolution of cast Ti/Zr-based bulk metallic glass composites (BMGCs) containing β-Ti still remains ambiguous. This is why to date the strategies and alloys suitable for producing such BMGCs with precisely controllable volume fractions and crystallite sizes are still rather limited. In this work, a Ti-based BMGC containing β-Ti was developed in the Ti-Zr-Cu-Co-Be system. The glassy matrix of this BMGC possesses an exceptional glass-forming ability and as a consequence, the volume fractions as well as the composition of the β-Ti dendrites remain constant over a wide range of cooling rates. This finding can be explained in terms of a two-phase quasi-equilibrium between the supercooled liquid and β-Ti, which the system attains on cooling. The two-phase quasi-equilibrium allows predicting the crystalline and glassy volume fractions by means of the lever rule and we succeeded in reproducing these values by slight variations in the alloy composition at a fixed cooling rate. The two-phase quasi-equilibrium could be of critical importance for understanding and designing the microstructures of BMGCs containing the β-phase. Its implications on the nucleation and growth of the crystalline phase are elaborated.

Bulk metallic glasses (BMGs) are metastable alloys, which are commonly produced through rapid solidification of metallic melts^1^,^2^,^3^,^4^. They generally exhibit high strengths, a large elastic limit, and good corrosion resistance because the lack of translational symmetry does not permit lattice defects such as grain boundaries or dislocations, which their crystalline counterparts usually contain^2^,^3^,^4^. However, BMGs generally do not exhibit any significant macroscopic plasticity in tension^5^,^6^ since the plastic deformation is highly localized in narrow regions termed shear bands^7^,^8^. In order to enhance the ductility, bulk metallic glass composites (BMGCs) composed of (a) soft crystalline phase(s) and a glassy matrix have been developed^9^,^10^,^11^,^12^,^13^,^14^,^15^,^16^,^17^,^18^,^19^,^20^,^21^,^22^,^23^,^24^,^25^,^26^,^27^. The ductile crystalline particles act as obstacles for shear bands and hence hamper the rapid propagation of a detrimental shear band. Instead, these heterogeneities often even multiply the number of shear bands^7^,^8^. Depending on the nature of the crystalline phase, the currently widely-studied *in-situ* BMGCs can be classified into two main groups^28^,^29^,^30^: (i) CuZr-based BMGCs containing B2 CuZr^10^,^11^,^12^,^13^,^14^,^15^,^16^, which precipitates during cooling and (ii) Zr/Ti-based BMGCs, in which β-Zr/Ti dendrites are embedded in a glassy matrix^17^,^18^,^19^,^20^,^21^,^22^,^23^,^24^,^25^,^26^,^27^. However, CuZr-based BMGCs have two drawbacks: they are relatively poor glass formers, which severely restricts the dimensions of the samples and, simultaneously, it is rather difficult to control the morphology (volume fraction, size and distribution of the B2 phase) of the microstructure^12^,^13^. Both factors negatively affect the reproducibility of microstructures and the respective mechanical properties^12^,^13^.

Similarly, the glass-forming ability (GFA) of the glassy matrices of Zr/Ti-based BMGCs is often also limited and the critical casting thicknesses are known to be generally below 20 mm^19^,^20^,^21^,^22^,^23^,^24^,^25^. More importantly, the volume fraction of the β-phase can be only adjusted in a relatively small range of cooling rates during Bridgman solidification^21^,^29^. An alternative route to tailor the microstructure is to modify the Be content of the glass-forming alloys, whose microstructure is then designed in the course of a semi-solid treatment^19^,^20^. Therefore, developing novel BMGCs comprising a glassy matrix with an exceptional high GFA is needed from a practical point of view and, simultaneously, a more advanced and detailed understanding of the solidification process in these particular BMGCs is required in order to produce novel BMGCs with custom-made microstructures.

In this work, a Ti_45.7_Zr_33.0_Cu_5.8_Co_3.0_Be_12.5_ (at.%) alloy was developed consisting of β-Ti dendrites embedded in a glassy matrix. The Be-rich matrix (Ti_32.02_Zr_30.13_Cu_9.01_Co_4.84_Be_24.00_) can congeal into glassy ingots with a weight of up to 150 g. A two-phase quasi-equilibrium between two metastable phases, i.e. the supercooled liquid and β-Ti dictates the compositions of both phases as well as the according volume fractions. Based on the lever rule, a series of BMGCs with precisely controllable fractions of β-Ti was successfully produced for alloys with crystalline volume fractions above 30%. The influence of the cooling rates on the microstructures of these BMGCs is also elucidated. The two-phase quasi-equilibrium has strong implications on designing and understanding the evolution of typical microstructures found in cast BMGCs containing the β-phase.

## Results

In order to avoid listing a vast number of multicomponent alloys with only slightly varying compositions, we will use a special abbreviation for all alloys investigated in this work. The sample name indicates the expected crystalline mole fraction. “BT48” for example represents a composition, which contains 48% mole of crystals and 52% mole of glass. This estimation is based on the lever rule for the quasi-equilibrium phase diagram [Fig f1][Fig f2](cf. Fig. 3a). This can then be converted into volume fractions (see methods). BT0 (Ti_32.02_Zr_30.13_Cu_9.01_Co_4.84_Be_24.00_) hence labels the composition of the glassy phase and BT100 represents the composition of the β-Ti dendrites (Ti_60.58_Zr_36.11_Cu_2.30_Co_1.01_) as determined by means of wavelength-dispersive X-ray spectroscopy (WDS, see Table 2). As we will see below the volume fraction of these alloys is constant for a wide range of cooling rates. Therefore we do not need to specify a particular cooling rate for these alloys.

### Microstructures of the alloy BT48 cast at different cooling rates

**Figure 1 f1:**
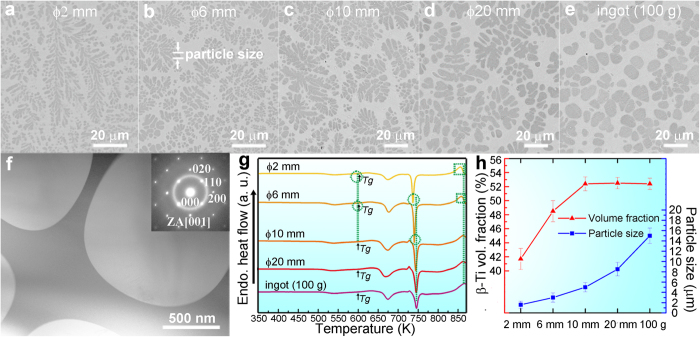
Characterizations of the samples of BT48 obtained at different cooling rates. (**a–e**) The back-scattered scanning electron microscopy (SEM) micrographs of the as-cast rods with diameters of 2 mm, 6 mm, 10 mm, and 20 mm, and the ingot of 100 g, respectively. The different cooling rates have a strong influence on the volume fractions, the shapes, and the size of β-Ti. (**f**) The transmission electron microscopy (TEM) micrograph of the rod with a diameter of 6 mm with an inset of diffraction pattern from the two-phase interface, showing a two-phase microstructure containing a glassy matrix and β-Ti with a body-centered cubic (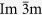) symmetry. (**g**) Differential scanning calorimetry (DSC) traces of the samples. The differences between the rods with a diameter of 2 mm or 6 mm and other samples are marked by circles (arising from the glassy matrices) and rectangulars (arising from β-Ti), indicating the compositions of both phases in the rods with a diameter of 2 mm or 6 mm are slightly different with other samples. The exothermic events at about 540 K before *T*_*g*_ arise from the metastable β-Ti. (**h**) The summary of the measured volume fractions and particle sizes (herein refer to the diameters of the secondary arms, as shown in (**b**)) of β-Ti in different samples. Error bars (s.d.) of the fluctuations of the measured volume fractions and particle sizes of β-Ti are given. Although particle sizes increase with lowering the cooling rates, the volume fractions of β-Ti are identical in the rods with diameters of 10 mm, and 20 mm, and the ingot of 100 g.

The microstructures of the as-cast rods of the alloy BT48 (Ti_45.7_Zr_33.0_Cu_5.8_Co_3.0_Be_12.5_) with diameters of 2 mm, 6 mm, 10 mm, and 20 mm, as well as of a 100 g ingot are shown in Fig. 1a–e, respectively. All five samples show a similar two-phase microstructure containing dendrites and a featureless matrix. The bright-field transmission electron microscopy (TEM) image of the rod with a diameter of 6 mm (Fig. 1f) depicts the dendritic phase and the matrix at a much higher magnification. Very sharp interfaces are visible and the selected area electron diffraction image can be indexed according to β-Ti. A halo is superimposed on the diffractions spots, which indicates the presence of an amorphous phase. The differential scanning calorimetry (DSC) traces of all samples are shown in Fig. 1g. A glass transition (marked with arrows) precedes the exothermic crystallization events and it can be concluded that the matrix is indeed glassy. At high temperatures (above 850 K) the endothermic transformation from α-Ti to β-Ti is detectable.

Figure 1a–e proves that the morphology of the dendrites changes with cooling rate. The faster the cooling, the finer the dendrites become. In the case of the ingot (Fig. 1e) the dendrite arms are almost circular in the cross-sectional cut. Based on these micrographs we analyzed the particle size and crystalline volume fraction as a function of cooling rate (Fig. 1h). In this work, “particle size” refers to the diameters of the secondary dendrite arms as indicated in Fig. 1b to facilitate the comparison. The average particle sizes gradually increases from 1.6 ± 0.7 μm (ϕ2 mm) to 3 ± 0.9 μm (ϕ6 mm) to 5 ± 0.9 μm (ϕ10 mm) to 8.5 ± 1.3 μm (ϕ20 mm) and eventually to 15 ± 1.5 μm in the case of the 100 g ingot. The volume fractions (for the conversion please see Methods) of β-Ti in the ϕ2 mm and ϕ6 mm rods are about 41.7 ± 1.5% and 48.5 ± 1.5%, respectively. While the crystalline volume fraction initially increases it remains constant within the experimental uncertainty for the rods with a diameter of 10 mm and 20 mm as well as for the 100 g ingot (52.5 ± 0.9 vol.%). This suggests that only coarsening of the β-Ti dendrites occurs above a critical cooling rate.

From secondary ion mass spectrometry (SIMS) conducted for the rod with a diameter of 20 mm (Supplementary Fig. 1), it is clear that the dendrites are Ti-rich, Cu– and Co-lean. The Be-signal in β-Ti is very week, suggesting that only a very small amount of Be is dissolved in β-Ti, a phenomenon commonly observed in Be-containing BMGCs^17^,^20^. Since it is impossible to accurately determine the content of a light element like Be in the glass or the dendrites by microscopic techniques, we shall assume in the following that all Be is dissolved in the glassy matrix. This approximation of course has an impact on the estimated compositions of the glass and the Ti dendrites. But the SIMS results imply that this error in composition should be rather small and, after all, should not affect the concept of a quasi-equilibrium we want to put forward here. The other elements dissolved in the dendrites were measured by means of electron probe micro analyzer (EPMA) with WDS for all samples excluding the 2 mm rod due to its small particle size. The values are listed in Table 1. The composition of β-Ti in the rods with a diameter of 10 mm and 20 mm as well as in the 100 g ingot are almost identical. The concentration of Ti and Zr is higher and that of Cu and Co is lower compared with the composition of β-Ti in the ϕ6 mm rod.

These compositional differences of the constituent phases in the case of the ϕ6 mm rod and the other samples also reflect in the DSC traces (Fig. 1g). The second crystallization event (~740 K) is about 3 K lower for the 6 mm rod compared to the 10 mm, 20 mm rods and the 100 g ingot. This indicates that the composition of the matrix in the 6 mm rod might be slightly different. In addition, the α → β transformation peak (~860 K) detected in the rod having a diameter of 6 mm shifts by about 4 K to lower temperatures (marked by a rectangular). A lower α-to-β transformation temperature generally indicates that the β-Ti dendrites in the 6 mm rod contain a slightly higher amount of β-stabilizers (Cu and Co) than the other samples^31^. This is indeed consistent with the EPMA results obtained for the other samples (Table 1). Similar differences are also observed for the DSC curve of the ϕ2 mm rod. However, the almost identical DSC traces of the larger rods (ϕ10 and ϕ20 mm) and of the 100 g ingot suggest that the glass and dendrites are chemically identical in these samples. The composition of the glassy matrix in the 100 g ingot was also obtained by EPMA, which does not allow for the concentration of Be (Table 1). The EPMA results was further verified by considering the mass conservation for each element:


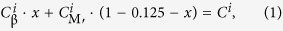


where 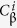, 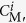, and 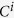 are the concentrations of each element *i* (*i*: Ti, Zr, Cu, Co) in β-Ti, in the glassy matrix and in the nominal composition, respectively. 0.125 and *x* are the Be content in the overall composition and the mole fraction of β-Ti, respectively, which has been measured from the micrographs in Fig. 1. The estimation based on all four elements (Ti, Zr, Cu and Co) yield very similar values with regard to the β-Ti mole fraction, *x*, in the 100 g ingot (Ti: *x* = 47.91%, Zr: *x* = 47.85%, Cu: *x* = 47.88%, Co: *x* = 48.02%). In other words, the EPMA results seem to be consistent. We used the mean value of *x* = 47.9% as the mole fraction of β-Ti phase, which is in good agreement with the measured volume fraction of 52.5 ± 1% (Fig. 1h). Applying the approximation discussed above we recalculated the composition of the glassy matrix by assuming that all Be is dissolved in the glass (Table 1). The estimated compositions of the glassy matrix and β-Ti are Ti_32.02_Zr_30.13_Cu_9.01_Co_4.84_Be_24.00_ (at.%, BT0) and Ti_60.58_Zr_36.11_Cu_2.30_Co_1.01_ (at.%, BT100), respectively.

**Figure 2 f2:**
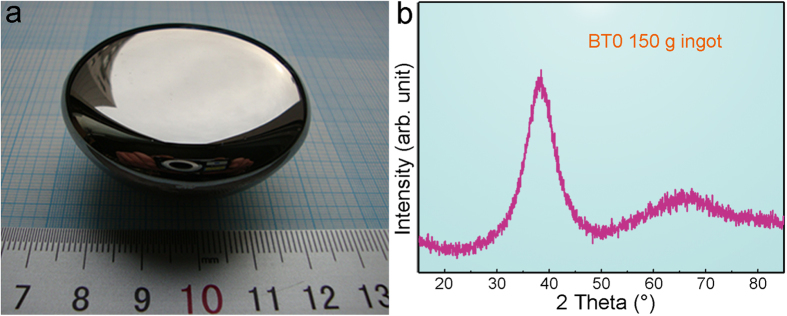
The as-solidified 150 g ingot. (**a**) The appearance of the 150 g ingot of Ti_32.02_Zr_30.13_Cu_9.01_Co_4.84_Be_24.00_ (at.%, BT0). The mirror-like surface and the diffraction humps in its X-ray diffraction pattern (**b**) indicate that it is fully amorphous.

The present results imply that the glassy matrix is an exceptional glass former because it does not crystallize even in the 100 g ingot and it even restricts the growth of β-Ti. In order to evaluate the glass-forming ability of BT0, a Ti_32.02_Zr_30.13_Cu_9.01_Co_4.84_Be_24.00_ ingot with a weight of 150 g was produced (Fig. 2a). It has a mirror-like surface and the corresponding XRD pattern (Fig. 2b) proves that it is fully amorphous. Because the cooling rate at which a 100 g ingot solidifies is equivalent to that of the cylinder with a diameter of 50 mm quenched in water^26^ or in a copper mould^32^, BT0 is one of the best BMG formers developed so far^26^,^32^,^33^,^34^,^35^,^36^, comparable to Vitreloy 1.

## Discussion

### Two-phase quasi-equilibrium

As suggested by Lin and Johnson^37^, the typical cooling rate during copper mould casting can be estimated by the following equation:


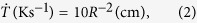


where *R* and 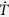 are the sample radius and the cooling rate, respectively. This estimation yields the following cooling rates for the various samples: 1 × 10^3^ Ks^−1^ (ϕ2 mm), 1.1 × 10^2^ Ks^−1^ (ϕ6 mm), 40 Ks^−1^ (ϕ10 mm), 10 Ks^−1^ (ϕ20 mm) and 1.6 Ks^−1^ (100 g ingot). It is known that the cooling rate usually has a strong influence on the volume fraction of the β-phase in Zr/Ti-based BMGCs and the volume fractions tend to increase with lowering the cooling rates^21^,^29^. Because the cooling rate in the 100 g ingot is more than 20 times lower than in the case of the rod with a diameter of 10 mm, there is much more time for the precipitation of β-Ti. Consequently, the crystalline volume fraction should be much higher in the ingot than in the ϕ10 mm rod. Contrary to expectation, the volume fraction of β-Ti does not change when the cooling rate is between 40 and 1.6 K s^−1^ and only the dendrites become coarser (Fig. 1h).

**Figure 3:**
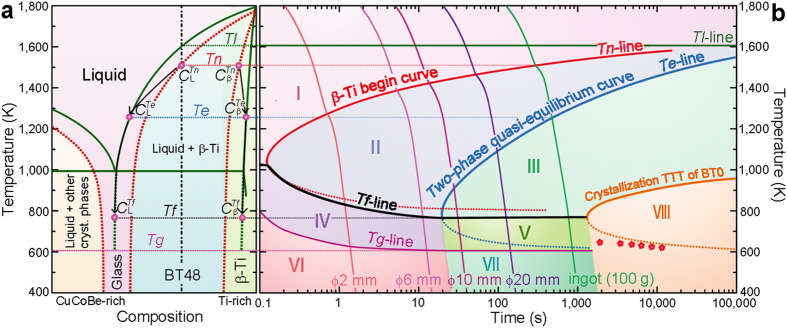
Schematic illustrations of the two-phase quasi-equilibrium. (**a**) Schematic binary phase diagram. The green solid lines represent the equilibrium liquidus line, the solution limit of β-Ti and the eutectic line. The left two red dashed lines describe the beginning stage at which β-Ti or other crystalline phases begin to precipitate in the undercooled melt at the cooling rate representing the ingot with a weight of 100 g. β-Ti begins to precipitate as the melt is cooled down and intersects the red dashed lines, and the composition of β-Ti is 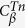. It is slightly richer in solute (Cu and Co) than its equilibrium composition (the far right green line). For higher cooling rates, the degree of undercooling increases^39^ and thus the red dashed lines shift to lower temperatures. As the fraction of β-Ti increase, the compositions of both phases change from 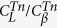 to 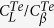. When the quasi-equilibrium is established between the (undercooled) liquid and β-Ti, the compositions of both phases change along the liquidus and the solution limit of β-Ti (green lines) from 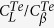 to 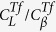. (**b**) Schematic continuous cooling transformation curves (CCT) for BT48. The β-Ti begin curve (*T*_*n*_–line), the two-phase quasi-equilibrium curve (*T*_*e*_–line), and the crystallization TTT curve of BT0 are drawn between their *T*_*l*_ and *T*_*g*_, respectively. (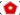) are DSC measured times before crystallization of BT0 plus (2000 K-990 K)/(1 K s^−1^). The cooling curves starting from 2000 K are indicated by the different sample diameters (calculated from equation (2)). Phases in regions with different colours are: I, liquid; II, liquid + β-Ti (non-equilibrium compositions, changeable microstructure); III, liquid + β-Ti (equilibrium compositions, changeable microstructure); IV, supercooled liquid + β-Ti (non-equilibrium compositions, unchangeable microstructure); V, supercooled liquid + β-Ti (quasi-equilibrium compositions, unchangeable microstructure); VI, non-equilibrium BMGCs; VII, quasi-equilibrium BMGCs; VIII, crystallized matrix + β-Ti. If cooling curves intersect *T*_*e*_–line (not crystallization curve of BT0), quasi-equilibrium BMGCs will be achieved, otherwise, non-equilibrium BMGCs will be obtained. The sketches of thermodynamic views are shown in Supplementary Fig. 2.

A possible mechanism to explain the current findings is to assume that the system attains a quasi-equilibrium between two metastable phases during cooling. Figure 3a depicts the schematic binary phase diagram for the present alloy system. The green solid lines indicate the equilibrium phase diagram. Next to the complex mixture of equilibrium phases (e.g.^32^ Be_2_Zr and Cu_10_Zr_7_) at Cu-Co-Be-rich compositions, only β-Ti and the liquid phase have to be considered. As observed for most glass-forming Zr/Ti-based alloys^34^,^36^, the present system is a eutectic one. Besides the equilibrium lines, the red dotted lines belonging to a metastable phase diagram are displayed in Fig. 3a. The left two red dashed lines describe the temperatures at which nucleation of β-Ti or the other crystalline phases (Be_2_Zr and Cu_10_Zr_7_) sets in. The degree of undercooling for a given composition is then determined by the temperature difference between the equilibrium liquidus line and the metastable liquidus line. For higher cooling rates, the degree of undercooling will increase and thus the red dashed liquidus lines will shift to lower temperatures. The red dotted line at high Ti concentrations reflects the solubility of Cu, Co and Be in β-Ti during fast cooling. Quenching of metallic melts generally results in supersaturation by solute trapping at the early stages of crystallization^38^,^39^ and therefore the red dotted line is placed left of the equilibrium solidus line (Supplementary Fig. 2).

At the specified composition BT48 (Fig. 3a), β-Ti begins to precipitate when the melt is cooled down and intersects the red dotted line at *T*_*n*_. The liquid’s composition is 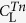 and that of β-Ti is 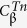. Growth of the β-phase nuclei is controlled by atomic diffusion^22^,^24^. When the temperature falls below *T*_*n*_, the volume fraction of β-Ti increases quickly due to the fast atomic diffusion in the slightly undercooled liquid. The contribution of the element *i* to the growth velocity, *g*^*i*^, is given by^40^:


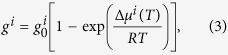


where 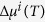 is the molar chemical potential difference of the element *i* between the undercooled liquid and crystalline phase (here: β-Ti), 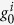 is a constant at temperature *T*, and *R* is the ideal gas constant. Equation (3) is generally applied to isothermal processes. Here, we regard the continuous cooling process during solidification as a sequence of short isothermal treatments at varying temperatures^39^. Therefore, equation (3) is believed to also describe the crystallization process under non-isothermal conditions. Atomic diffusion is pronounced at high temperatures and thus the volume fraction of β-Ti should increase quickly. This is accompanied by a relatively fast change of the composition of the supercooled liquid from 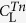 to the equilibrium value, 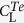. Simultaneously, the dendrites alter their composition from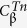 to the equilibrium composition, 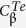 (Fig. 3a), which effectively lowers the undercooling of the melt. The composition-dependent chemical potential in both phases also changes and reduces the driving force for crystallization, 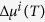. There exists a temperature, *T*_*e*_, where the driving force for the precipitation of β-Ti vanishes:


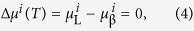


i.e. the chemical potentials of element *i* in both phases (supercooled liquid and β-Ti) are equal, and the system establishes a two-phase equilibrium (Supplementary Fig. 2). At *T*_*e*_, the compositions 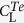 and 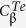
of both phases are located on the equilibrium lines of the phase diagram (green lines in Fig. 3a). Below the eutectic temperature, the glass should decompose into various crystalline phases (e.g.^32^ Be_2_Zr and Cu_10_Zr_7_) if the system is given sufficient time. Due to the relatively fast cooling, which we apply here, this process is suppressed as is the transformation of β-Ti to α-Ti. These two metastable phases are retained and the green dotted lines describe their compositional changes on further cooling. Since diffusion is rather limited at these relatively low temperatures, these lines are rather steep (Fig. 3 a). This is also a result of the negligible changes in the Gibbs free energy differences of both phases at very low temperatures^41^. Once a certain temperature, *T*_*f*_, is crossed, the compositions of the constituent phases can be assumed constant and temperature-independent. As a final step, the supercooled liquid vitrifies at the glass transition temperature, *T*_*g*_.

The resulting microstructure consists of two metastable phases and therefore we use the term “quasi-equilibrium” in this context. The mole ratio of both phases should then be governed by the lever rule and the composition of the supercooled liquid, which then transforms into a glass, should be 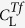. This corresponds to alloy BT0 (Ti_32.02_Zr_30.13_Cu_9.01_Co_4.84_Be_24.00_). Two-phase equilibria are usually only established for phases in their equilibrium states^39^. However, a two-phase quasi-equilibrium can also occur between two metastable phases when their chemical potentials are identical. Metastable phase diagrams have been used to understand the formation of metastable phases via solidification^42^ or solid-state amorphization^43^, for instance. Lee *et al.*^44^ have also constructed a pseudo-binary equilibrium phase diagram by measuring the melting points of several Zr-based BMGCs containing β-Zr and used the metastable phase diagram to explain the evolution of the different microstructures in their samples. However, they did not address the influence of the cooling rate on volume fractions and compositions as well as they did not focus on the underlying mechanism. The current concept of a two-phase quasi-equilibrium successfully reveals the influence of cooling rates and designed mole fractions of β-Ti on the microstructures of BMGCs as we discuss below.

### Continuous cooling transformation (CCT) curves

The precipitation of β-Ti in the present system can be captured by continuous cooling transformation (CCT) curves. We use the quasi-equilibrium phase diagram to construct a CCT curve for the present alloys (Fig. 3b). It is also the basis for discussing the differences in the crystalline volume fractions and compositions of the glass and β-Ti for BT48 samples produced with different cooling rates (see above). The following temperatures are crucial here: *T*_*l*_ (the equilibrium liquidus temperature), *T*_*n*_ (the temperature when nucleation commences), *T*_*e*_ (the temperature when the compositions of the supercooled liquid and β-Ti reach the equilibrium values), *T*_*f*_ (the temperature when diffusion stops and the composition are effectively frozen in), *T*_*g*_ (the glass transition temperature) and finally the temperatures at which the supercooled liquid crystallizes into Be_2_Zr and Cu_10_Zr_7_. The cooling curves (constant cooling rate given by equation (2)) for the rods having a diameter of 2 mm, 6 mm, 10 mm, and 20 mm as well as the cooling rate for the 100 g ingot of BT48 are displayed (Fig. 3b).

CCT curves are difficult to obtain, and are often calculated from isothermal temperature-time-transformation (TTT) curves by the Grange and Kiefer method^45^,^46^. Due to the resemblance of CCT curves and the upper parts of TTT curves^47^, TTT curves are widely used to schematically describe the crystallization process of a melt during continuous cooling^2^,^4^,^47^. TTT curves of alloys with various GFA were experimentally constructed by Johnson *et al.*^48^, and the “nose” temperature (*T*_*nose*_) of the C-shaped curve has been found to be linked to the glass transition temperature, *T*_*g*_: *T*_*nose*_  ≈ 1.3 *T*_*g*_^48^. Due to its propensity to precipitate β-Ti, BT48 is a poor glass former (*T*_*g*_/*T*_*l*_ ≈ 0.5) and between its equilibrium liquidus temperature (*T*_*l*_ = 1546 K) and its glass transition temperature (*T*_*g*_ ≈ 773 K), the *T*_*n*_ curve with a nose temperature *T*_*nose*_ ≈ 1.3 *T*_*g*_ ≈ 1040 K can be drawn. Similarly, the temperatures at which the glassy matrix (with the composition BT0) crystallizes into Be_2_Zr and Cu_10_Zr_7_ among other phases^32^ (orange curve in Fig. 3b) is also drawn between *T*_*l*_ = 990 K and *T*_*g*_ = 602 K with a *T*_*nose*_ ≈ 1.3 *T*_*g*_ = 783 K. The measured times are modified by adding (2000 K – 990 K)/(1 Ks^−1^) for the comparison with the cooling curves from 2000 K, because the critical cooling rate of BT0 for glass formation is estimated about 1 K s^−1^. Now that the different phase fields are established, the solidification behaviour under different cooling rates can be discussed qualitatively.

The cooling curves corresponding to the BT48 rods with a diameter of 2 mm and 6 mm intersect the *T*_*n*_–line but not the *T*_*e*_–line and thus go through the phase regions I, II, IV, and VI (Fig. 3b). Once the *T*_*n*_–line is crossed β-Ti precipitates in the supercooled liquid. Before the composition of the supercooled liquid or the dendrites reaches the equilibrium composition, *T*_*f*_ is traversed and the compositions and volume fractions are frozen in due to the limited mobility of atoms. At the glass transition temperature the supercooled liquid finally vitrifies and a BMGC is the outcome. In this cooling rate regime the composition and the volume fractions depend on the cooling rates. For this reason one obtains identical phases in case of rod with 6 mm in diameter but dendrites are Ti-richer and the glass is Ti-poorer compared to the rods with smaller diameters (ϕ2 mm). At the same time the crystalline volume fraction is somewhat larger because the system has a larger time window for β-Ti to precipitate and the dendrite size increases as well. In contrast, the cooling curves representing the larger rods (ϕ10 mm and ϕ20 mm) as well as the 100 g ingot of BT48 intersect the *T*_*n*_– and *T*_*e*_–lines, and go through regions I, II, III, V, and VII (Fig. 3b) before the microstructure evolution is effectively halted at *T*_*f*_. This in turn results in BMGCs in which the composition of the Ti dendrites and the glassy matrix and consequently also the respective volume fractions reach a quasi-equilibrium (Fig. 3a). Owing to the steep slopes near *T*_*f*_ (Fig. 3a) the compositions of β-Ti and the supercooled liquid remain virtually constant near *T*_*f*_. The same holds for the volume fractions because of the conservation of mass. Instead, the main microstructural change in region III is the coarsening of the dendrites driven by the interfacial energy, known as Ostwald ripening^20^,^22^,^39^. The cylindrical arm with a smaller diameter has a higher chemical potential. During the precipitation of β-Ti dendrites, the outer parts of the dendrite arms have larger diameters than the inner root parts. During ripening the root parts of β-Ti dissolve and then re-precipitate on the head parts, leading to a more particle-like morphology. Similarly, smaller particles have a higher chemical potential than the larger ones, causing large particles to become larger and more spherical, and smaller ones to become smaller and eventually to vanish^39^. In other words, the lower cooling rates mean more time for ripening of the dendrites. The ripening process in region III also provides the possibility to design BMGCs with various shapes and sizes of β-Ti (without altering the volume fractions) by simply changing the cooling rates in this regime.

### Producing BMGCs with precisely controlled volume fractions of β-Ti

In order to test our hypothesis of a quasi-equilibrium for the present alloy system, we have synthesized a variety of alloys with compositions ranging from BT0 up to BT95 (Table 2). If the quasi-equilibrium as depicted in Fig. 3a exists than for a rod diameter above 10 mm, the volume fraction of the glassy phase and β-Ti should be predictable by applying the lever rule. The composition of the glass should be Ti_32.02_Zr_30.13_Cu_9.01_Co_4.84_Be_24.00_ (BT0) and that of the dendrites Ti_60.58_Zr_36.11_Cu_2.30_Co_1.01_ (BT100). Therefore, the crystalline mole fraction, *x*, in the BMGCs should be given by:


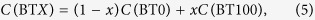


where 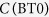 and 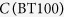 are the compositions of the glassy matrix (BT0) and β-Ti (BT100), respectively. These alloys are abbreviated BT*X* (*X* = 100*x*) and their compositions are listed in Table 2. The expected volume fractions, 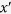, can be calculated from the mole fractions, *x*, by:


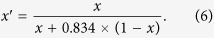


**Figure 4:**
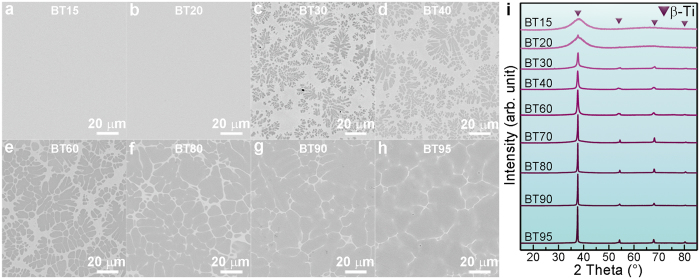
Characterization of as-cast BT*X* rods with a diameter of 10 mm. (**a–h**) The back-scattered scanning electron microscopy (SEM) micrographs of BT15, BT20, BT30, BT40, BT60, BT80, BT90, and BT95, respectively. BT15 shows a fully amorphous structure, and other samples have a two-phase microstructure. The growing intensities of the diffraction peaks (110) of β-Ti from BT20 to BT95 suggest an increasing volume fraction of β-Ti. (**i**) The X-ray diffraction patterns of BT*X*. The compositions of BT*X* are designed using the lever rule and calculated by equitation (5), as listed in Table 2. The designed mole fractions, *x*, of β-Ti have a strong influence on the final microstructure and the measured volume fractions of β-Ti are listed in Table 2.

The details on the deduction of equation (6) can be found in the Methods section, and the calculated 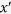 are also listed in Table 2. The microstructures for the alloys BT15 to BT95 found in rods with a diameter of 10 mm are displayed in Fig. 4a–h. The crystalline volume fractions were extracted from these micrographs. While for BT15 and BT20 only a featureless micrograph is revealed, all other alloys exhibit the typical composite microstructure consisting of dendrites embedded in a featureless matrix. The crystalline volume fractions clearly increase with increasing *x*. Starting from BT30 there is a satisfactory agreement between the estimated crystalline volume fractions and the measured values (Table 2). The alloys BT10 up to BT15 are fully amorphous and apparently the lever rule does not describe the volume fractions properly. Obviously, the precipitation of β-Ti is aggravated as the composition of BT*X* shift towards BT100, due to the composition containing less Be, Cu and Co, which stabilize the undercooled liquid^33^,^34^,^36^,^43^. This means the *T*_n_–line as depicted in Fig. 3b is shifted to larger times for these compositions and the entire melt hence vitrifies at the given cooling rate. Additionally, the compositions of β-Ti for *x* ≥ 0.40 were measured by means of energy-dispersive X-ray spectroscopy attached in SEM and were found to be similar to Ti_60.58_Zr_36.11_Cu_2.30_Co_1.01_ (BT100).

The XRD patterns of the as-cast rods with a diameter of 10 mm of various BT*X* alloys are shown in Fig. 4i. As *x* ≤ 0.15, no crystalline diffraction peaks were detected in the 10 mm rod of BT15, indicating a fully amorphous structure. Weak reflections of β-Ti begin to become detectable in the 10 mm rod of BT20 and its intensities increase with increasing *X.* This suggests an increasing volume fraction of β-Ti. These results are corroborated by TEM micrographs (Supplementary Fig. 3), which prove the fully glassy nature of the BT15 rod with a diameter of 10 mm and in the ϕ10 mm rod of BT20 TEM reveals the presence of small β-Ti dendrites.

**Figure 5:**
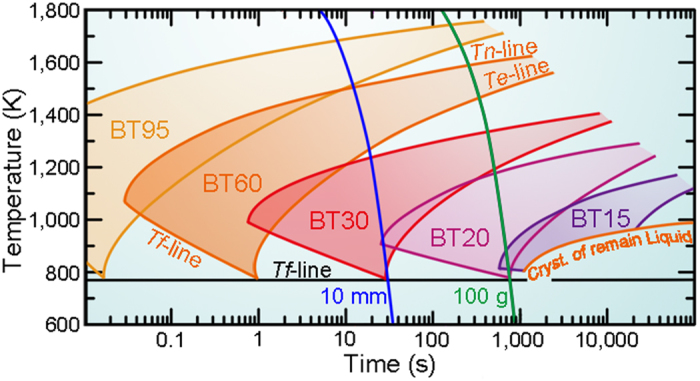
Influence of designed mole fractions, *x*, on the two-phase quasi-equilibrium. The precipitation of β-Ti is more difficult as *x* becomes smaller and the liquidus temperature also decreases, causing the *T*_*n*_–lines and *T*_*e*_–lines to shift to larger times and lower temperatures. The *T*_*n*_–lines, *T*_*e*_–lines, and *T*_*f*_–lines of BT95, BT60, BT30, BT20, and BT15 and the crystallization curve of the remaining liquid of BT15 are drawn. The cooling curves (indicated by the diameter or the weight of the samples) of the as-cast rod with a diameter of 10 mm and the ingot of 100 g obtained by equation (2) are also plotted. There are two cooling-rate dependent critical values: 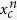 and 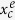. If the cooling curves do not intersect the *T*_*n*_–line (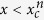), fully amorphous are the outcome. If the cooling curves intersect the *T*_*n*_–line but not the *T*_*e*_–line (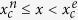), non-equilibrium BMGCs will be obtained; if the cooling curves intersect the *T*_*e*_–line (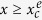), quasi-equilibrium BMGCs will be achieved, and then the fraction of β-Ti can be precisely controlled by the lever rule. In the as-cast rods with diameters of 10 mm of current BT*X*, 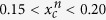, 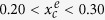.

Finally, the CCT curves for the BT*X* shall be discussed (Fig. 5). For several alloys the *T*_*n*_–, *T*_*e*_– and *T*_*f*_–lines are drawn. Two cooling rates are also depicted, one for a rod with a diameter of 10 mm and for an ingot with a weight of 100 g. As *x* ≥ 0.3, the cooling curve of the rod intersects the *T*_*e*_–lines of these alloys and the outcome is a microstructure, which has attained a two-phase quasi-equilibrium: The compositions of both phases are identical in all alloys and crystalline volume fraction can be predicted (Table 2). As *x* = 0.2, however, the cooling curve of the 10 mm rod crosses the *T*_*n*_–line at a temperature close to its nose temperature, and then quickly reaches the *T*_*f*_–line, generating a non-equilibrium BMGCs with a very small fraction (<5%) of β-Ti (Table 2). When *X* is even smaller (e.g. *X* = 15), the cooling curve of the 10 mm rods does not cross the *T*_*n*_–line and a fully glassy rod can be obtained. At slower cooling (100 g ingot of BT15), yet, β-Ti precipitates with a volume fraction less than 5% (Fig. 2a and Supplementary Information) because the cooling curve representing the 100 g ingot intersects the *T*_*n*_–line (Fig. 5). Since the cooling rate of the 100 g ingot approaches the critical cooling rate for the formation of a glassy matrix, BT10 and BT15 can never achieve the quasi-equilibrium BMGCs due to decomposition of the supercooled liquid into Be_2_Zr and Cu_10_Zr_7_ at even lower cooling rates.

The discussion above unravels that there are two cooling-rate dependent critical β-Ti mole fractions: 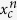 and 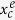. As 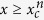, the cooling curves cut through the *T*_*n*_–lines and as 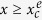, the cooling curves intersect the *T*_*n*_– and *T*_*e*_–lines. At the same cooling rate, fully amorphous states, “non-quasi-equilibrium” BMGCs, and “quasi-equilibrium” BMGCs can be achieved as 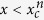, 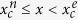, and 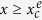, respectively. In the current as-cast 10 mm rods of BT*X*, 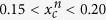 and 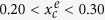. Both 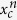 and 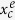 decrease with lowering the cooling rate.

In summary, a Ti-based BMGCs containing β-Ti was developed in the Ti-Zr-Cu-Co-Be system, and its glassy matrix can solidify into fully glassy ingots up to 150 g. A two-phase quasi-equilibrium is established during continuous cooling and determines the microstructural evolution in terms of composition of the constituent phases as well as their volume fractions. Also its dependence on the cooling rate can be understood by assuming this quasi-equilibrium between two metastable phases. The nature of the two-phase quasi-equilibrium and a steep liquidus line near the temperature at which diffusion is effectively suppressed, *T*_*f*_, account for the identical volume fractions and compositions of β-Ti in the rods with a diameter of 10 mm, 20 mm rods as well as in the 100 g ingot of the alloy BT48 (Ti_45.7_Zr_33.0_Cu_5.8_Co_3.0_Be_12.5_). The influence of designed β-Ti mole fractions on the microstructures of BMGCs can also be understood based on the two-phase quasi-equilibrium. By carefully modifying the composition the respective volume fractions of the composite can be predicted and composite microstructures can thus be tailored. Moreover, the size of the dendrites can be adjusted by choosing the appropriate cooling rate for a given alloy while the volume fractions remain constant. In this way, one has a large degree of freedom in designing BMGCs. We envision that the two-phase quasi-equilibrium has scientific significance for the understanding of BMGCs with various microstructures.

## Methods

### Sample preparation

The 100 g ingots of all alloys listed in Table 2 and the 150 g ingot of BT0 were prepared by arc melting Ti, Zr, Cu, Co, and Be pure metals (purities are over 99.9 wt. %) under a Ti-gettered high-purity argon atmosphere. As the chemical homogeneity was achieved after re-melting four times, the melts solidified into semi-spherical ingots within the water-cooled copper crucibles. The as-cast rods with diameters of 2 mm, 6 mm, 10 mm, and 20 mm of BT48 and the as-cast rods with diameters of 10 mm of other BT*X* were prepared by copper-mould casting after re-arc melting the master ingots in high-purity argon atmosphere.

### Characterization

The specimens were transversely cut from the as-cast rods and from the upper central part (the lowest cooling rate) of the 100 g or 150 g ingots for the characterization by means of X-ray diffraction (Philips PW1050, Cu-Kα), scanning electron microscopy (Zeiss Supra 55) combined with energy-dispersive X-ray spectroscopy (Oxford) and differential scanning calorimetry (DSC, Netzsch 204F1) at a constant heating rate of 20 K min^−1^. The liquidus temperatures of BT0 and BT100 were measured claorimetrically (Netzsch 404). The DSC isothermal time before crystallization was obtained by heating at rates of 60 K min^−1^ to and then holding at different temperatures between 610 K and 640 K using a Netzsch 204F1. The elemental mapping of the 20 mm rod of BT48 was carried out by secondary ion mass spectroscopy (ION-TOF, SIMS5). The composition of the constituent phases in BT48 samples cooled at different rates were quantitatively acquired by an electron probe micro analyzer (Shimadzu 1610) with a wavelength-dispersive spectroscopy (WDS). The microstructure of the ϕ6 mm rod of BT48, the ϕ10 mm rods of BT15 and BT20 were investigated in a transmission electron microscope (FEI Tecnai F20) with an electron energy loss spectrum (EELS) system attached. The TEM samples were prepared by ion-milling with the cooling of liquid nitrogen using a Gatan 691.

### Measurement of volume fractions and particle sizes of β-Ti

We used the software Image J (http://imagej.nih.gov) to directly measure the area fraction of β-Ti from the SEM micrographs^12^,^25^. Supposing the area fraction of β-Ti in the cross section at height of *h* in a unit cubic is *A*(*h*), then the volume fraction can be obtained by the relation: 
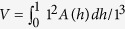
. In the current BMGCs, β-Ti precipitates within the liquid by homogenous nucleation which is then followed by diffusion-controlled growth. Thus β-Ti is distributed homogeneously on the micrometer-scale in the BMGCs, causing that the *A*(*h*) slightly fluctuates near the measured average volume fraction 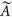. In this case 
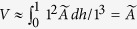
. This method shows good accuracy, because the fluctuations of *A*(*h*) are quite small (within 1.5%, seen in Fig. 1h). Particle sizes were also measured using Image J by directly measuring the diameters of secondary arms or particles of β-Ti. We also estimate the volume fractions of β-Ti in the 10 mm rod of BT20 and the 100 g ingot of BT15 by comparing their intensities of diffraction peak (110) of the XRD patterns with that of BT95, i.e.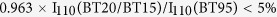.

### Converting mole fraction *x* into volume fraction *x’*

For convenience we accept that there is one mole atoms of BMGCs and the mole fraction of β-Ti phase is *x*. The volume of β-Ti and glassy matrix (BT0) are given by 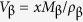 and 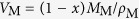, respectively, where 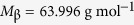 and 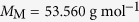 are the molar weight of BT100 and BT0 (listed in Table 1), respectively. 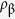 and 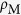 are densities of single-crystal β-Ti and BT0, respectively. 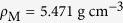 was obtained by the Archimedes method, and 
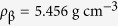
 was calculated using 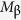 and XRD measured volume size 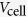 of the unit cell of β-Ti by 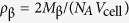. The relationship between *x* and 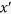 is given by 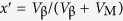, and then equation (6) was obtained by substituting corresponding values.

## Additional Information

**How to cite this article**: Zhang, L. *et al.* Two-phase quasi-equilibrium in β-type Ti-based bulk metallic glass composites. *Sci. Rep.* **6**, 19235; doi: 10.1038/srep19235 (2016).

## Supplementary Material

Supplementary Information

## Figures and Tables

**Table 1:** Chemical compositions of the constituent phases in the samples BT48 cooled at different rates.

Samples	Phases	Compositions
6 mm	β-Ti	Ti_59.51_Zr_35.16_Cu_3.49_Co_1.84_
10 mm	β-Ti	Ti_60.59_Zr_36.13_Cu_2.27_Co_1.01_
20 mm	β-Ti	Ti_60.54_Zr_36.17_Cu_2.29_Co_1.00_
	β-Ti	Ti_60.58_Zr_36.11_Cu_2.30_Co_1.01_
100 g	glassy matrix	Ti_42.12_Zr_39.65_Cu_11.86_Co_6.37_
	glassy matrix	Ti_32.02_Zr_30.13_Cu_9.01_Co_4.84_Be_24.00_>*

The estimated compositions of the glassy matrix and β-Ti in the 100 g ingot are the quasi-equilibrium compositions and are denoted BT0 (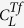) and BT100 (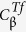), respectively. *This composition is calculated from the EPMA obtained compositions and the nominal composition by assuming that Be is totally in the glassy matrix.

**Table 2:** The compositions, designed β-Ti volume fractions, and measured β-Ti volume fractions in the as-cast 10 mm rods of BT*X*.

Alloys No.	Mole Fraction x (%)	Compositions (at.%)	Expected Vol. Fraction x’ (%)	Measured Vol. Fraction (%)
BT0	0	Ti_32.02_Zr_30.13_Cu_9.01_Co_4.84_Be_24.00_	0	0
BT10	10	Ti_34.88_Zr_30.73_Cu_8.34_Co_4.46_Be_21.60_	11.8	0
BT15	15	Ti_36.30_Zr_31.03_Cu_8.00_Co_4.27_Be_20.40_	17.5	0
BT20	20	Ti_37.73_Zr_31.33_Cu_7.67_Co_4.07_Be_19.20_	23.1	<5
BT30	30	Ti_40.59_Zr_31.92_Cu_7.00_Co_3.69_Be_16.80_	33.9	35.3 ± 2
BT40	40	Ti_43.44_Zr_32.52_Cu_6.33_Co_3.31_Be_14.40_	44.4	45.1 ± 1.5
BT48	47.9	Ti_45.7_Zr_33.0_Cu_5.8_Co_3.0_Be_12.5_	52.4	52.5 ± 1.0
BT60	60	Ti_49.16_Zr_33.72_Cu_4.98_Co_2.54_Be_9.60_	64.3	65.0 ± 1.0
BT70	70	Ti_52.01_Zr_34.32_Cu_4.31_Co_2.16_Be_7.20_	73.7	74.3 ± 0.9
BT80	80	Ti_54.87_Zr_34.91_Cu_3.64_Co_1.78_Be_4.80_	82.7	83.2 ± 0.8
BT90	90	Ti_57.72_Zr_35.51_Cu_2.97_Co_1.39_Be_2.40_	91.5	91.6 ± 0.8
BT95	95	Ti_59.15_Zr_35.81_Cu_2.64_Co_1.20_Be_1.20_	95.8	96.3 ± 0.9
BT100>*	–	Ti_60.58_Zr_36.11_Cu_2.30_Co_1.01_	–	–

*BT100 is the quasi-equilibrium composition of β-Ti in the 100 g ingot of BT48.
