# Supplementary material for: Two-phase quasi-equilibrium in β-type Ti-based bulk metallic glass composites
Source: Sci Rep. 2016 Jan 12;6:19235. doi: 10.1038/srep19235 (PMC4709698; doi:10.1038/srep19235)
Supplement: Supplementary Information [file srep19235-s1.doc]

**Two-phase quasi-equilibrium in β-type Ti-based bulk metallic glass composites**

L. Zhang^1,2^, S. Pauly^2,*^, M. Q. Tang^1^, J. Eckert^2,3^, H. F. Zhang^1,*^

^1^Shenyang National Laboratory for Materials Science, Institute of Metal Research, Chinese Academy of Sciences, 110016 Shenyang, China

^2^IFW Dresden, Institute for Complex Materials, P.O. Box 27 01 16, D-01069 Dresden, Germany

^3^TU Dresden, Institute of Materials Science, D-01062 Dresden, Germany

Correspondence and requests for materials should be addressed to H.F.Z. (email: [hfzhang@imr.ac.cn](mailto:hfzhang@imr.ac.cn)) or to S.P. (email: [s.pauly@](mailto:s.pauly@)ifw-dresden.de).


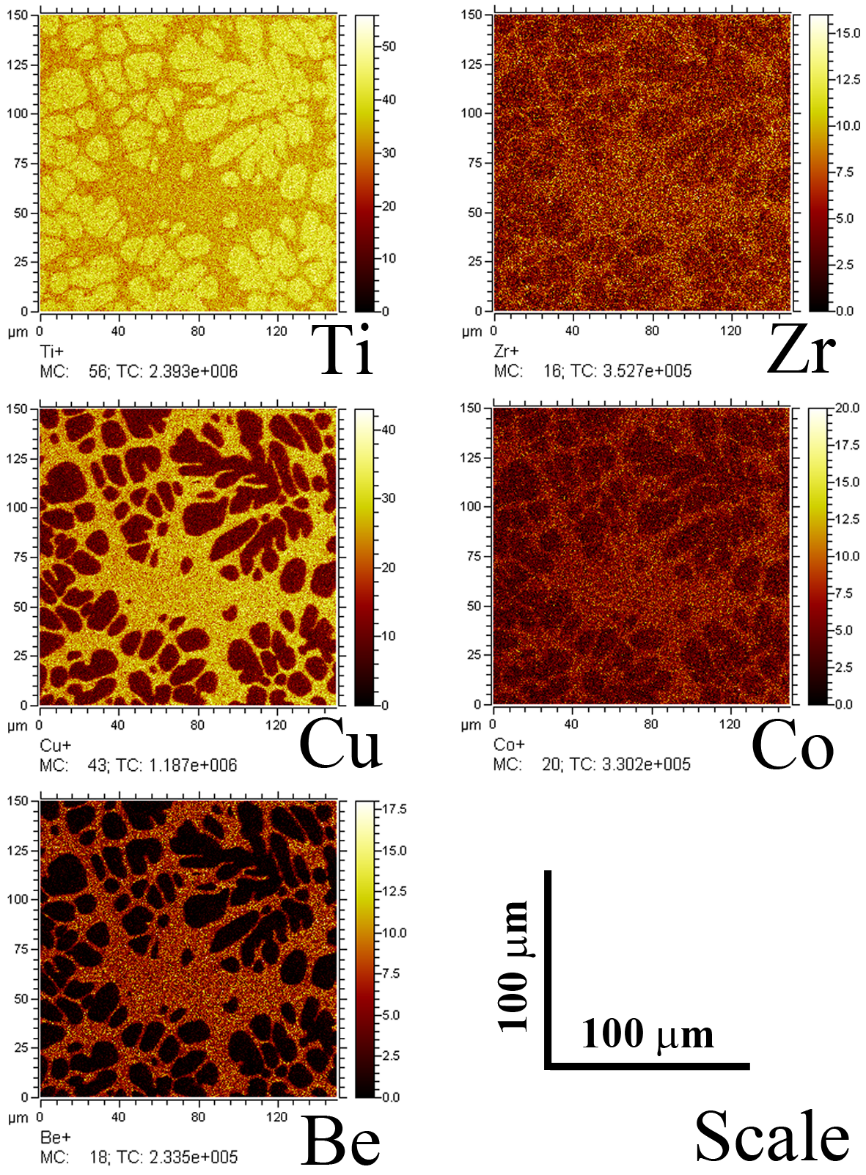
**Supplementary Figure 1.** **The secondary ion mass spectrometry (SIMS) obtained elemental mappings of the as-cast rod with a diameter of 20 mm of BT48.** β-Ti phase is Ti-rich, Cu- and Co-lean. The signal of Be in β-Ti is very weak, and it is almost fully black (~0 at.%), suggesting just a very small amount of Be can be detected within β-Ti. However, at the same time, the Be signal is quite strong in the glassy matrix, and therefore, it is reasonable to assume that Be is totally dissolved in the glassy matrix^1^. All constituent elements are distributed homogenously within either phase.


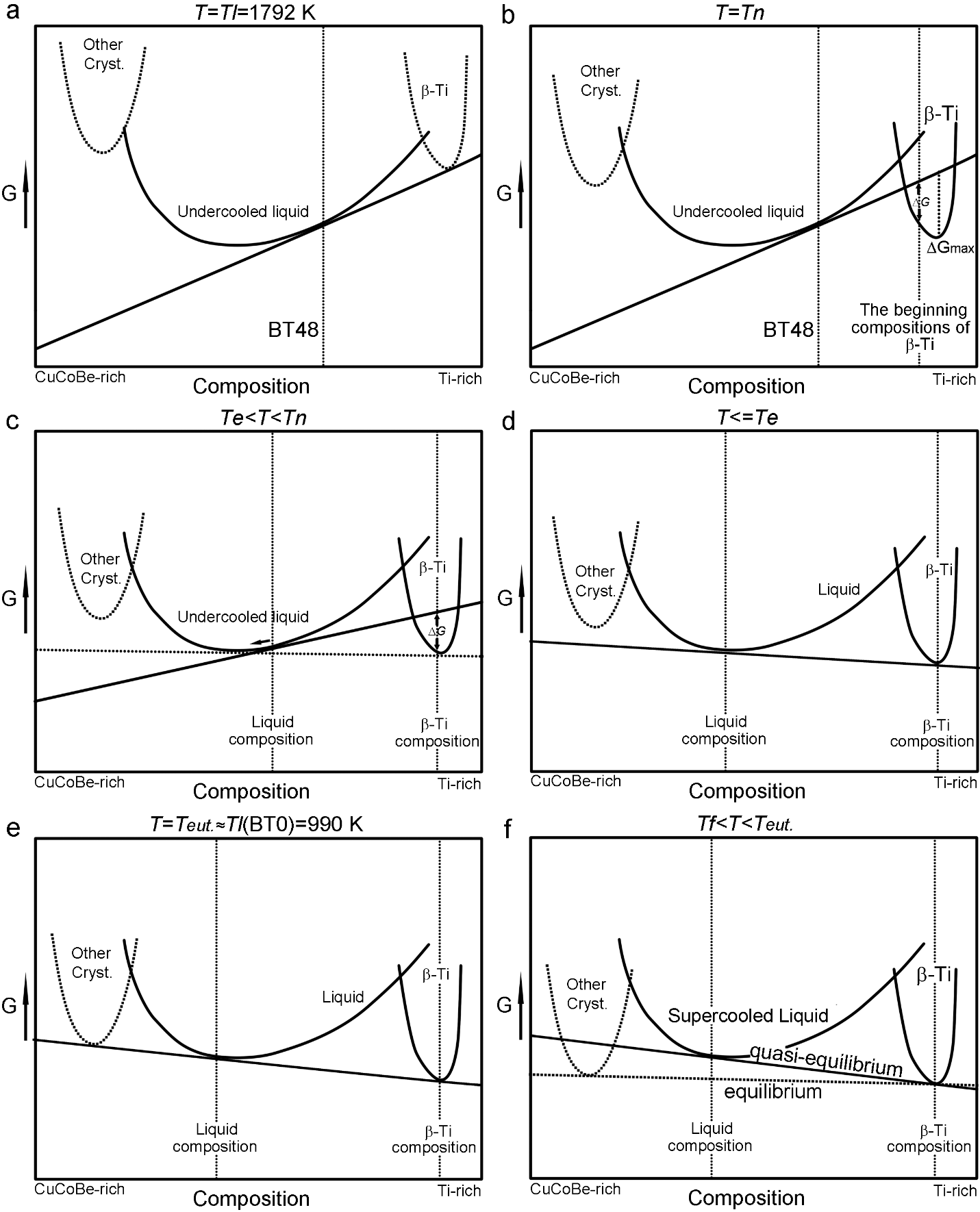
**Supplementary Figure 2. Schematic sketches of thermodynamic views at different temperatures of BT48 during continuous cooling.** β-Ti begins to precipitate at the temperature *T_n_* at a cooling rate of the 100 g ingot, and its beginning composition $C_{\beta}^{Tn}$ is deviated from the one where the driving force is the maximum, due to the solute trapping^2,3^, as a result, $C_{\beta}^{Tn}$ (closer to $C_{L}^{Tn}$) is more kinetically favored, as shown in (b). As the fraction of β-Ti increases, the composition of liquid will shift towards the equilibrium composition (tangent point), as indicated by the arrow in (c). Because the fast diffusion of atoms at high temperature, both phases begin to be in the two-phase quasi-equilibrium at temperature *T_e_*. The atomic diffusion can catch up the change of thermodynamic states of both phases, keeping the two-phase quasi-equilibrium at temperatures below *T_e_*. As the temperature below the eutectic temperature *T_eut._*, other crystalline phases are thermodynamically favored to form. However, the large degree of undercooling of the metastable liquid is needed to overcome the nucleation barrier for the precipitation of other crystalline phases (Cu_10_Zr_7_ and Be_2_Zr) during continuous cooling, and the two-phase quasi-equilibrium between β-Ti and metastable liquid are still maintained (f). Until the temperature near or below *T_f_*, the increasing viscosity of the liquid results in that the microstructure does not change any more, and finally, is frozen into BMGCs at *T_g_* of BT0.


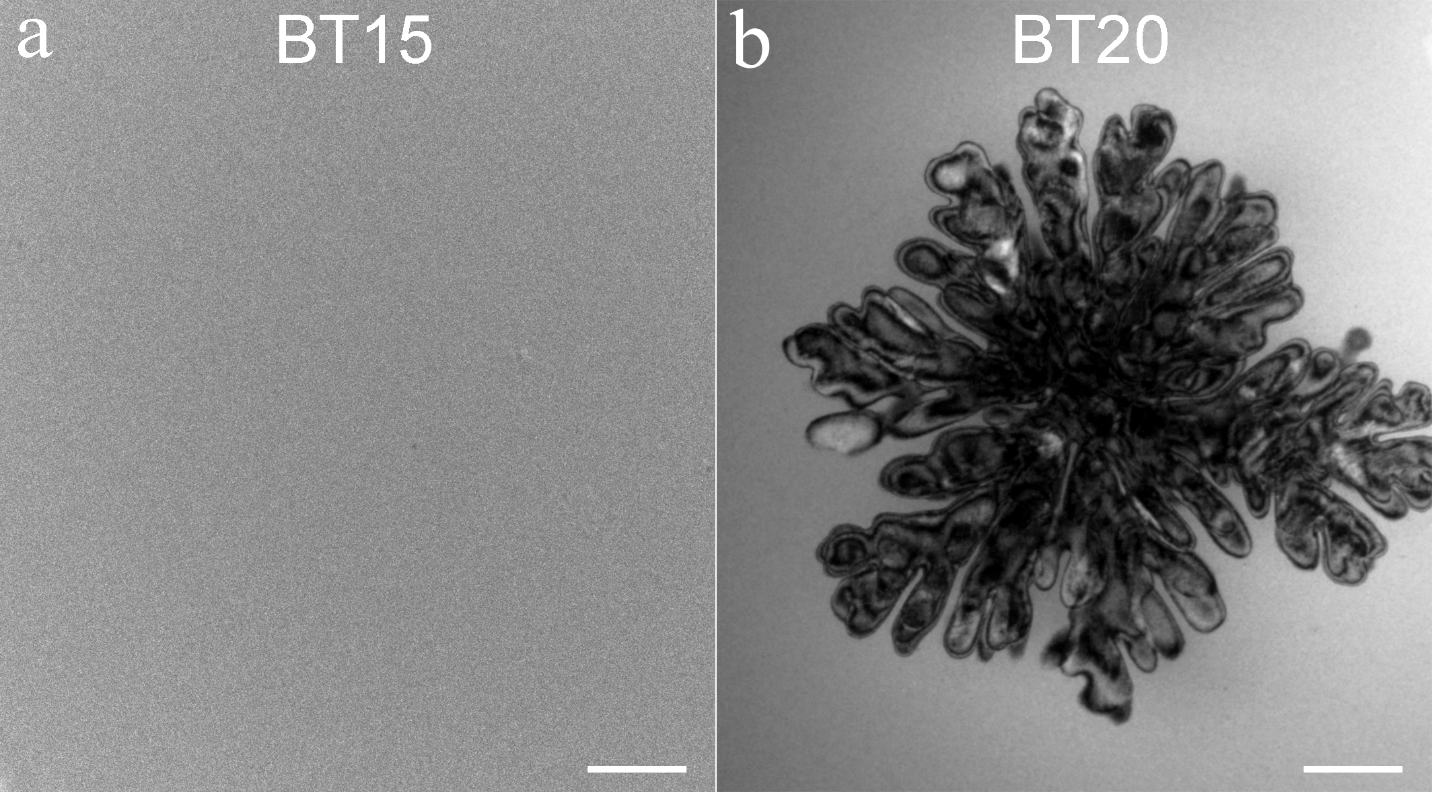
**Supplementary Figure 3. Transmission electron microscopy (TEM) micrographs of the as-cast rods with a diameter of 10 mm of BT15 (a) and of BT20 (b).** Scale bars are 200 nm. The featureless micrograph of BT15 suggesting a fully glassy nature. Tiny dendrites of β-Ti in BT20 could be observed on its TEM micrographs (the volume fraction of β-Ti is estimated less than 5%, see in the **Methods**).


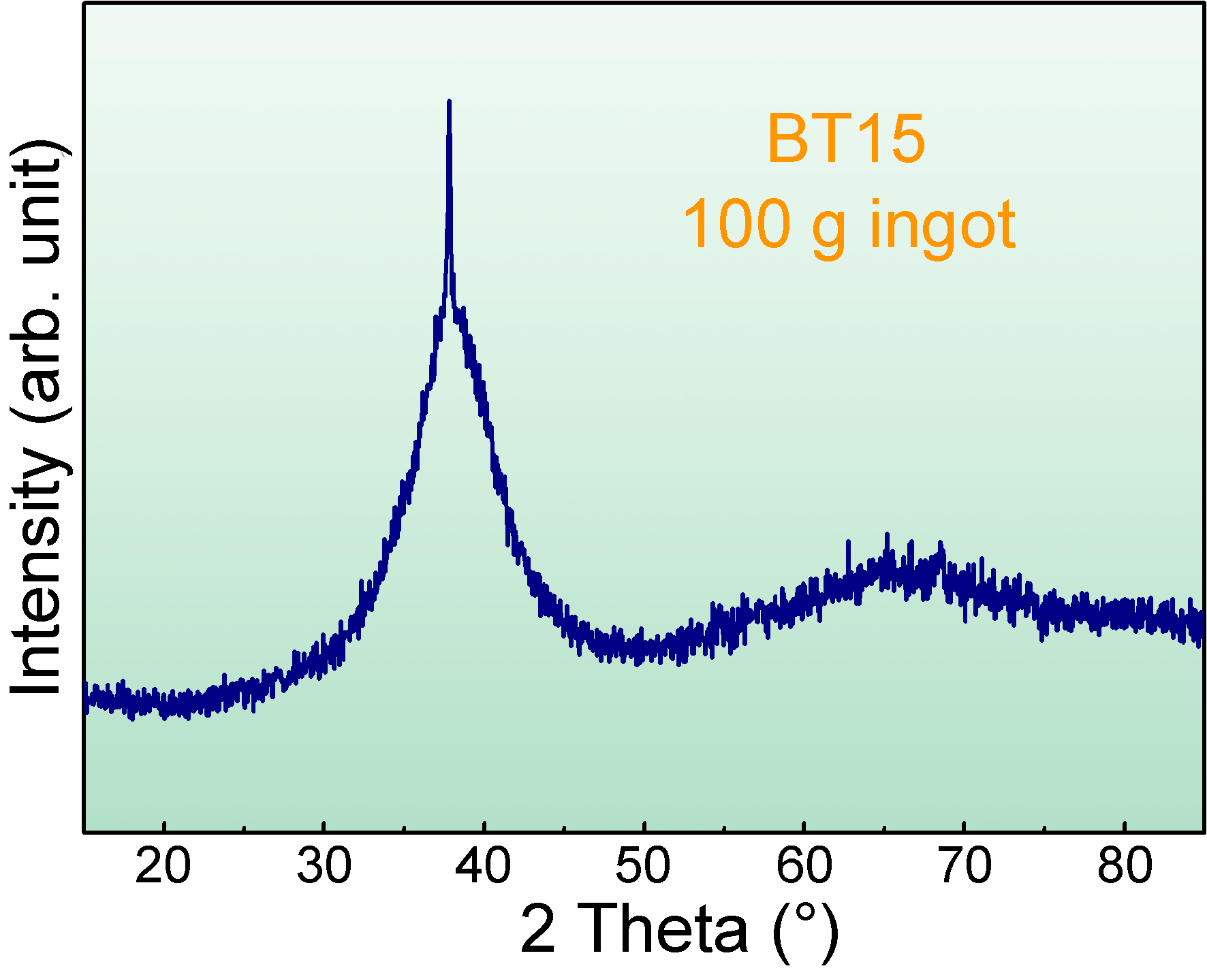
**Supplementary Figure 4. The X-ray diffraction pattern of the 100 g ingot of BT15.** The diffraction peak (110) of β-Ti is imposed on the diffuse hump of the glassy matrix, indicating a two-phase microstructure. The volume fraction of β-Ti in the 100 g ingot of BT15 is estimated less than 5% (see in **Methods**).

**References**

1. Hofmann, D. C. et al. Designing metallic glass matrix composites with high toughness and tensile ductility. Nature 451, 1085-1089 (2008).

2. Baker, J. C. & Gahn, J. W. Solute trapping by rapid solidification. *Acta Metall.* **17,** 575-578 (1969).

3. Kurz, W. & Fisher, D. J. in *Fundamentals of solidification 3rd edn*, Appendix 6, 220-221 (Trans Tech Publications, 1992).
